# Is there a Chinese pattern of the second demographic transition?

**DOI:** 10.1007/s42379-022-00113-0

**Published:** 2022-09-10

**Authors:** Jia Yu, Yu Xie

**Affiliations:** 1 Peking University, Beijing, China; 2 Princeton University, Princeton, USA

**Keywords:** The second demographic transition, Chinese pattern, Family changes, Marriage and childbearing

## Abstract

The Second Demographic Transition (SDT) is a useful theoretical framework for explaining the recent trend in many countries of very low fertility combined with alternative union and family types. Although past studies have observed the SDT in many Western societies, whether it is applicable to East Asia remains unclear. Capitalizing on data from the Chinese Census and China Family Panel Studies, we provide estimates of key behavioral and ideational indicators of the SDT. We find that union formation in China has trended increasingly toward patterns commonly observed in the West, including delayed age of marriage and the common practice of premarital cohabitation. While having a lowest-low fertility rate, China has not experienced rising nonmarital childbirths, a key component of the SDT. However, we observe growing tolerance toward nonmarital childbearing and childlessness. Marriages remain relatively stable in China, especially among couples with children. Taken together, our analysis suggests that typically coincident changes in patterns of family behavior associated with the SDT are not occurring simultaneously in China. Moreover, ideational changes are preceding behavioral changes, particularly in attitudes toward nonmarital childbearing and childlessness. Our research suggests a different pattern of the SDT in China, which has been heavily influenced by Confucian culture.

## Introduction

In the past century, the First Demographic Transition (FDT)—declines in mortality followed by declines in fertility—has swept through most societies in the world (Casterline, 2003). When the FDT theory was first proposed, it was predicted that fertility decline would stop at the replacement level so that a population would remain stationary thereafter (Lee, 2003). However, sustained below-replacement-level fertility has been observed in Western and Northern European societies since the 1970s, and several new pathways to family formation have become prevalent, such as premarital cohabitation and nonmarital childbearing. To understand these newly emerging demographic phenomena, Lesthaeghe & van de Kaa, (1986) proposed the Second Demographic Transition (SDT) theory, and interpreted the SDT in different societal settings (van de Kaa, 1987; Lesthaeghe, 1995; Lesthaeghe et al., 2006, 2009; Surkyn & Lesthaeghe, 2004). As summarized by Coleman, 2007; p.402), “the second demographic transition, following hard on the heels of the first, describes and explains the revolution in living arrangements and sexual behavior, and in the setting for childbearing, now transforming the lives of many inhabitants of Western societies and, it is argued, eventually in developed societies elsewhere.”

Comparing FDT with SDT, the conceptual model of “Ready, Willing and Able” (RWA) proposed by Coale, (1973) is an excellent explanatory framework (Lesthaeghe & Vanderhoeft, 2001). According to the model, the adoption of new family behaviors must comply with three preconditions: adopting the new behavior must improve the overall utility of the actor (R); the new behavior must be accepted legally, morally, and culturally (W); actors are able to access to the innovations that facilitate the practice of new behavior (A). During the FDT, changes in production mode and the establishment of a formal education system led to the fact that having more children will not increase the overall family utility (R), and as people gradually accept the idea of contraception (W), fertility declines when contraceptive methods become widespread (A). In the FDT, the realization of W is relatively easier, making it often overlooked. However, in the SDT, the role of W has been emphasized more. In the Post-Industrial Era, R is not only about material satisfaction, but also includes the “higher-order” needs such as self-actualization and individual autonomy. Some new family behaviors like childlessness, divorce, and cohabitation would bring greater emotional satisfaction. Sexual revolution, gender egalitarian movement, the state's retreat, and other social changes have provided the necessary conditions (A) for people to adopt the new family behaviors. Under such circumstances, whether or not a family transition will occur depends primarily on the cultural and normative acceptance (W) of the new family behaviors. And this is the reason that the SDT emphasizes more on ideational changes than the FDT.

The notion of the SDT has been under debate since its inception. Some scholars contend that the SDT is merely a continuation of the classic demographic transition, and that the newly observed demographic developments are secondary features of the FDT (Cliquet, 1992; Coleman, 2004). In addition, other scholars believe that the SDT is archetypical of Western European (and also Canadian and Australian) societies but will not spread to the United States or Southern, Central, or Eastern Europe, let alone to Asia (for a review, see Lesthaeghe, 2010). In response, Lesthaeghe, (2010) argued that the driving forces of the SDT were different from those of the FDT, with ideational factors and women’s empowerment being particularly important. The SDT can also be distinguished from the FDT by its multifaceted manifestations. Beyond fertility, the SDT entails multiple family-related behaviors that are radically different from traditional practices of marriage and childbirth.

In terms of the applicability of the SDT framework, recent empirical studies have affirmed that the SDT is taking place in the US, Eastern Europe, and South America (Esteve et al., 2012; Lesthaeghe, 2010; Lesthaeghe & Neidert, 2006; Rotariu, 2006; Zakharov, 2008). However, due to data limitations, studies of the SDT in East Asian societies remain limited to date. Scholars usually pay attention to one or two indicators of the SDT, and no study has yet provided a quantitative assessment of all indicators of the SDT in an East Asian country (Raymo et al., 2015).

China has completed the FDT over recent decades (Cai, 2010; Wang, 2011). As a result of improved healthcare, for example, the life expectancy in China significantly increased from 42.2 for males and 45.6 for females in 1950 to 74.6 and 78.4 respectively in 2010 (Cai, 2013; Wang, 2011). Family planning policies implemented by the government during the early 1970s made the most significant contribution to fertility decline in China, with the total fertility rate falling from 5.8 in 1970 to 2.8 in 1977 (Cai & Wang, 2021). Later, the One-Child Policy was formally launched in 1980, further decreasing the fertility rate until it was below replacement level by the early 1990s (Wu, 2010). Due to economic development and ideational changes since the economic reform that began in 1978, the Chinese family has also undergone changes during the FDT, including reduced family size and delayed age of first marriage (Cai & Wang, 2021; Yu & Xie, 2015b). Stepping into the twenty-first century, more profound family changes associated with the SDT have been observed in recent studies, including a declining marriage rate, an increasing premarital cohabitation rate, and an increasing divorce rate (Mu & Xie, 2014; Wang & Zhou, 2010; Yu & Xie, 2021; Zeng & Wang, 2000). In addition, according to National Bureau of Statistics of China, the total fertility rate in 2020 declined to 1.3, signaling that China has entered the category of a lowest-low-fertility society. Nevertheless, it is still under debate whether the SDT is really taking place in China. On the one hand, some signature family behaviors of the SDT remain rare in China, for example, nonmarital childbearing (Yu & Xie, 2021). On the other hand, the presence of the ideational foundations of the SDT in China has not been well examined.

As suggested by the RWA model, ideational changes (W) would be the most crucial foundation for identifying the process of the SDT. In today’s China, the preconditions of R and A have been fairly met, but it is still unknown if there are substantial ideological shifts in family culture.

In this paper, capitalizing on data from the Chinese Censuses, a nationally representative survey, and an online survey, we contribute to the literature by providing an overall assessment of the SDT in China, including both the ideational and behavioral changes in the Chinese family. Moreover, situating our study in China, an East Asian society with a long tradition of Confucianism and a socialist political system, we ask whether or not there is an identifiably Chinese pattern of the SDT, with an affirmative answer indicating that the trajectories of the SDT may vary depending on cultural and political contexts.

## The Chinese family in transition

### The historical Chinese family

Traditionally, the family was considered the most important institution in Chinese society. People’s daily activities, such as agricultural production, intimacy, procreation, care work, and education, were mostly dependent on the family (Ebrey, 2003, 2014; Fei, 1946; Whyte, 1996, 2005). As the intermediate collectivity between the emperor and individuals, family lineages also helped with the imperial court’s administration. In addition, gender asymmetry was a prominent feature of the traditional Chinese family. Daughters were only transitory members of their natal families. After marriage, women belonged to their husband’s family (Greenhalgh, 1985; Parish & Willis, 1993). Below, at the risk of oversimplification, we provide a brief summary of family behaviors in traditional Chinese society in terms four key features.

First, the male head of the family lineage had ultimate authority over individual family members (Cohen, 1990; Hamilton, 1990; Stacey, 1983; Zheng, 2001). In Confucianism, personal sacrifice for the prosperity of the family was considered a virtue, and the family head carrying the familial lineage was primarily responsible for other family members’ behavior (Earley, 1989; Winfield et al., 2000). Moreover, the governance of the Chinese empire depended greatly on local family organizations for more than two thousand years (Fei & Liu, 1982; Leung & Nann, 1995). The family system administered most civil and legal affairs. In localities, the largest family clans had the authority to resolve civil disputes and allocate family-owned land. Such families were also obligated to provide collective services, such as building irrigation projects and sponsoring private schools (*si shu*) (Dutton, 1992; Freedman, 1961; Parish & Whyte, 1978; Sommer, 2000). According to their own family rules, family clans had the right to punish family members, including at rare and extreme circumstances sentencing them to death, without the approval of the state. As a result, individuals had to obey the arrangements of family clans in their economic activities, social lives, and family behaviors in ancient China.

Second, early and universal marriage was widely practiced in traditional Chinese society (Coale, 1992; Hajnal, 1982). The primary purpose of sexuality was procreation rather than recreation (Pan, 2007). The parent–child relationship was the most important relationship within the family, surpassing the conjugal relationship (Fei, 1992). Therefore, it was not up to the individual to decide whom to marry, rather parents would arrange marriages (Riley, 1994; Xu & Whyte, 1990). Parents would choose the most suitable spouse for a child in consideration of the overall interests of the family (Pimentel, 2000). Premarital sexual behavior was strictly forbidden to maintain the purity of the family bloodline, especially for women (Sommer, 2000; Theiss, 2004; Wang & Yang, 1996).

Third, to continue the bloodline, reproduction was the most important function of the family, especially giving birth to sons (Murphy et al., 2011; Poston, 2002). A woman could be legally expelled from her conjugal family due to infertility, and men could marry more than once if their first wife did not bear sons (Hong et al., 1993). Having more sons was crucial to Chinese families for several reasons: sons could enhance agricultural production by performing farming activities requiring heavy labor (Das Gupta et al., 2003); parents relied on their sons for their care during their elderly years (Sun, 2002); and in light of frequent conflicts between different family lineages over land, water, and other resources in some Chinese regions, sons were also preferred for their ability to contribute to the protection of the family and its property/resources (Cohen, 1968). A small fraction of ambitious families invested in sons’ education so that they might achieve official positions by passing the imperial examinations (*ke ju*) and in doing so promote the upward mobility of the whole family (Miyazaki, 1981; Wang, 2012).

Finally, women were in a subordinate position in the Chinese family system (Zuo, 2009). According to Confucianism, women owed obedience to three rotating male owners (*san cong*): “As a child, the girl belongs to her father; as a wife, to her husband; when the husband dies, she obeys her sons.” In traditional Chinese society, women’s activities in a well-respected family were limited within the inner residential quarters of the traditional homestead (Ebrey, 1993). Women could neither receive formal education nor sit for the imperial examinations, let alone occupy important positions in society (Bernhardt, 1999; Hinsch, 2010; Wang, 1999). Women had no right to initiate divorce, and remarriage of widowed women was discouraged (Mann, 1987; Palmer, 1995). In sum, Chinese women suffered great disadvantages in education, property ownership, and participation in public affairs.

### Changes in the Chinese family

Beginning with the 1911 Republican Revolution that overthrew the last imperial dynasty, the Qing, China experienced multiple social revolutions and transitions in the twentieth century, particularly the Communist Revolution that culminated in the founding of the People’s Republic of China in 1949, and the Cultural Revolution between 1966 and 1976. As a result, the traditional functions and authority of the family were deeply eroded by many formal social institutions. The *danwei* (work unit) system was established soon after the founding of the People’s Republic of China in 1949, and it defined the urban citizens’ social, economic, and political lives (Walder, 1986). On the one hand, workers and their families were dependent upon their *danwei* for material resources; on the other hand, the heads of *danwei* were given the authority to reward and punish *danwei* workers on behalf of the state (Xie et al., 2009). As a mediating collectivity between the state and individuals, *danwei* assumed some of the all-encompassing role of the traditional family in China, as individuals were now less restrained by their families.

Government policy and legislation have influenced Chinese people’s family behavior directly. After the Chinese government implemented family planning policies in the early 1970s, the fertility rate in China quickly began to decline (Cai, 2010; Gu et al., 2007). Combining with economic development and ideational changes, the One-Child Policy between 1980 and 2013 further reduced fertility to below-replacement levels after 1990 (Cai, 2013; Whyte et al., 2015). Legal regulations disrupted the tradition of early marriage. The first Marriage Law of the People’s Republic of China, promulgated in 1950, prescribed the legal marriage age for men and women to be 20 and 18 respectively, and marriage to a child bride was forbidden. As an accompaniment to the era’s stringent family planning policies, the legal marriage age was later postponed to 22 and 20 years of age for men and women respectively in the revised Marriage Law of 1980. The 1950 Marriage Law also abolished arranged marriages, with love-based matches being encouraged by the state. Thus, an individual’s preference began to play a prominent role in marriage formation (Davis & Friedman, 2014). Moreover, after 1950 all Chinese women had the right to initiate a divorce from their husbands, and widowed women were allowed to remarry. The government has also changed its treatment of non-traditional family behaviors. For instance, the revised Marriage Law of 1980 referred to unmarried cohabitation as “illegal cohabitation,” whereas a 2001 amendment to the law changed the wording to “nonmarital cohabitation” so that the negative connotation of cohabitation became neutral.

The rapid economic development spurred by the economic reform since 1978 has ushered in new changes to family structures and individual family behaviors in China. Due to the urbanization that has accompanied economic growth, many rural-to-urban migrants have been physically separated from their parents and now live independently in urban areas. As a result, parents’ authority over their children’s family lives has been significantly weakened. Without parental supervision, young adults have more freedom to choose lifestyles and experiment with novel behaviors such as premarital sex and unmarried cohabitation (Rosenfeld & Kim, 2005). In addition, parental influence on individual mate choice has been reduced. Marketization and rising inequality have changed the economic foundations of marriage in China, and economic prospects have begun to exert a significant influence on marriage entry (Mu & Xie, 2014; Yu & Xie, 2015b). Thus, marriage has become more of a social privilege than a universal practice common to all. Exposure to Western culture has also changed the traditional family ideologies in China. With growing material satisfaction, individualism has emerged and gradually replaced collectivism among the youth (Davis, 2014; Hansen & Svarverud, 2013; Yan, 2009, 2010). As a result, family behaviors have become less vulnerable to the judgments of others, and Chinese people in general have become more tolerant of traditionally unaccepted behaviors such as premarital sex, unmarried cohabitation, and divorce (Farrer, 2014).

In fact, even before the economic reform, China’s patriarchal and patrilineal family model had been challenged by the empowerment of women. During the Mao Zedong era, women’s liberation was one of the most significant social movements advocated by the government. Chinese women were encouraged to participate in the labor market as breadwinners for the family. The female labor force participation rate in China was one of the highest globally (Maurer-Fazio et al., 2011). As a result, Chinese women gained economic autonomy within the family and began to reject traditional gender role specializations. Women’s education has also significantly improved since 1949. In the early stages of the People’s Republic of China, mass campaigns to improve literacy rates granted women the opportunity to receive a formal education. In 1956, more than 20 million girls were enrolled in primary school, which was unprecedented in China’s history (Zhang, 1984). After the economic reform, education in China experienced further growth, especially in terms of college expansion around 2000. According to recent statistics, women’s average educational attainment has exceeded that of men in the youngest cohorts, with women surpassing men in both college and graduate education (Treiman, 2013; Wu & Du, 2018; Wu & Zhang, 2010). The improvement in Chinese women’s status in education and work has changed women’s attitudes toward marriage and the family. The economic independence of women makes marriage no longer a necessity. Moreover, to achieve a successful career, many highly educated women may postpone their entry into marriage and motherhood (Ji, 2015; Piotrowski & Tong, 2016).

### Continuity of the Chinese family

Rapid modernization and ideational shifts in recent decades have not completely eradicated the influence of traditional family culture in China. The concept of family lineage continues to carry considerable meaning to many Chinese people. As shown by recent studies, the majority of Chinese people still agree that individuals have the obligation to make the whole family glorious and continue the bloodline (Yu, 2021). As a result, children remain the core element of families in today’s China. Childlessness is looked upon unfavorably in less developed regions in China (Zhang, 2006). Indeed, various fertility intention surveys in China invariantly reveal that few Chinese people intend to be childless (Hou, 2015; Yu et al., 2020; Zheng et al., 2009; Zhuang et al., 2014), and the childlessness rate among married couples has stayed at a very low level (Yu & Xie, 2021). Unlike in the SDT in Western societies, in which many women choose to forgo motherhood, married women in China are still expected to bear children.

The great importance attached to children among the Chinese is also reflected in the high expectations for children’s education and upward mobility. This too has remained invariant up to the present day; among young people born in the 1980s and 1990s, a large majority of them think it is important to have promising children (Lei & Shen, 2015; Yu, 2021). What is particularly remarkable is that parents’ expectations for their children’s education are high across the entire spectrum of family socioeconomic status (SES), in contrast to the West, where expectations for children are closely correlated with family SES (Li & Xie, 2020). Many Chinese parents’ happiness and the fulfilment of their ambitions depend on whether their children are able to move up the social ladder and thereby achieve upward social mobility for their parents (Chyi & Mao, 2012; Mitchell, 2010). As in other East Asian societies with longstanding Confucian cultures, children in China are viewed as the private property of the extended family, and raising children is primarily the responsibility of the whole family rather than the state (Tang & Dong, 2006; Zeng & Xie, 2014; Zhang & Xie, 2016). Such beliefs are echoed in the high educational expenditures of Chinese families (Chi & Qian, 2016; Qian & Smyth, 2011). In this respect, many Chinese parents remain committed to maximizing their efforts to provide a better environment for their children, including maintaining marital stability. Therefore, we do not expect a substantial increase in the prevalence of nonmarital childbearing and divorce in China in the future.

In sum, today’s Chinese family is influenced by traditional Confucian culture, the legacies of the socialist revolution, and rapid modernization. Although the patriarchy and the authority of the family have declined, certain functions of the traditional Chinese family persist, especially in terms of attitudes toward childbearing and childrearing. Thus, while individualized family behaviors such as cohabitation and marriage entry have gained more acceptance, changes in family behaviors pertaining to childbearing and childrearing have been much less significant and much slower paced. To capture the overall trends in family changes in China, the first aim of our study is to provide a careful and comprehensive investigation of the SDT indicators. In addition, past research on family changes in China has primarily focused on behaviors, overlooking values and beliefs associated with the SDT. Our study further contributes to the literature by examining the attitudes toward childlessness, nonmarital childbearing, divorce, and other elements of the SDT among the Chinese. Finally, based on the empirical evidence, we will discuss whether the concurrent continuity and change in the Chinese family system constitutes a Chinese variant of the SDT.

## Data and measures

### Data

The data used for this study come from three sources. First, we use the 2020 China Census to calculate the mean age at first marriage. Second, we use China Family Panel Studies (CFPS) data to calculate the main indicators of the SDT, including cohabitation rate, divorce rate, nonmarital childbearing rate, and so on. CFPS is a nationally representative longitudinal survey of Chinese communities, families, and individuals, launched in 2010 by the Institute of Social Science Survey (ISSS) of Peking University (Xie & Hu, 2014). The 2010, 2012, 2014, 2016, 2018, and 2020 waves of CFPS were pooled together to construct a sample containing longitudinal information that is up to date for the most recent wave. In addition to detailed information about marriage and fertility, CFPS includes questions about attitudes toward various family behaviors and fertility intention. Finally, we use the data of the Chinese Intention and Behavior of Childbearing and Parenting Survey (CIBCPS) as a supplement. CIBCPS is an online longitudinal survey launched by the Center for Social Research at Peking University, aiming to collect data on attitudes and behaviors relating to marriage, fertility, and parenting among people aged between 18 and 50. We use the baseline data of CIBCPS collected in 2021 to analyze attitudes toward some SDT-associated behaviors, such as nonmarital childbearing.

### Measures

Data on age of first marriage collected by the 2020 Census were used to measure the period mean first marriage age. Premarital cohabitation experience is measured by a binary variable based on a question in CFPS that asked about the cohabitation experiences of adult respondents. Divorce is measured by the survival time of marriage. CFPS contains the marital history of each adult respondent, through which we construct the marriage duration variable. Fertility and marriage information from the CFPS was used to construct variables indicating premarital childbearing and childlessness.

In previous studies, scholars usually measured values and beliefs relating to the SDT with the SDT index, including attitudes toward unmarried cohabitation, voluntary childlessness, nonmarital childbearing, and divorce with young children. However, none of the Chinese surveys incorporated such a standard module. Therefore, we utilize similar questions in different surveys to explore ideational changes associated with the SDT in China. For childlessness, we construct two measures of intended childlessness and attitudes toward it. The ideal number of children is included in the CFPS data, and we define persons whose desired number of children is zero as those who intend to be childless. We also use the attitude toward the statement “motherhood makes a woman complete” in CFPS to measure tolerance of childless women. In CIBCPS, respondents were asked if they agree that “single women have the right to give birth to children,” which is used to measure attitudes toward nonmarital childbearing. In CFPS, parents were asked if they agree that “parents should never divorce for the sake of their children, even in an unhappy marriage,” and we use this as a proxy for approval of divorce with young children. Unfortunately, neither CFPS nor CIBCPS include questions about attitudes toward cohabitation. Yet as premarital cohabitation is already being widely practiced by Chinese youth (Yu & Xie, 2017), we assume its high tolerance in today’s China.

To capture trends in family changes, we focus on differences across birth cohorts born in eight successive periods: (1) before 1950, (2) between 1950 and 1959, (3) between 1960 and 1969, (4) between 1970 and 1979, (5) between 1980 and 1984, (6) between 1985 and 1989, (7) 1990 and 1994, and (8) 1995 and 2002. To facilitate comparison, we combine some of the birth cohorts in selected analyses. Changes in diverse SDT indicators across the birth cohorts reveal the trends of family changes and the developmental path of the SDT in China. In addition, we have used the cross-sectional weights provided by the CFPS for all the descriptive statistics.

## Results

### Marriage entry

**Fig. 1:**
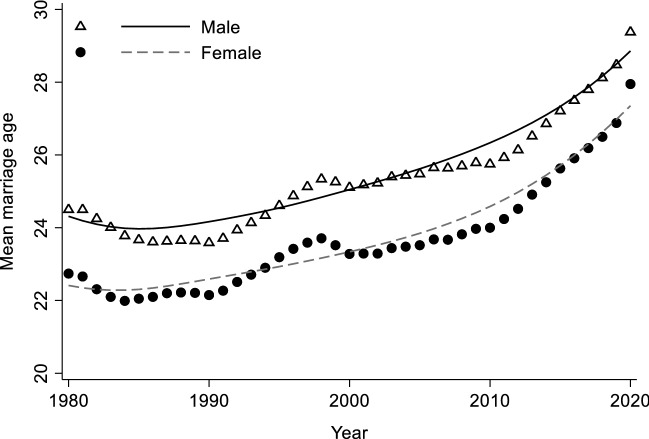
Mean age of first marriage by year for chinese men and women. Data source: 2020 China Census

Figure 1 shows the trends in average first marriage age for Chinese men and women. We observe a progressive rise in first marriage age for both men and women. From 1980 to 2019, the mean first marriage age increased from 24.5 to 28.5 for men and 22.7 to 26.9 for women. On average, the age of first marriage was delayed by one year per decade. Partly due to the outbreak of Covid-19, the average first marriage age experienced a drastic increase in 2020 in China, reaching 29.4 for men and 28.0 for women, approaching the level of other East Asian countries such as Japan and South Korea (Yu et al., 2020). However, it will require more time to determine whether age at first marriage will continue to rise or will fall back after the pandemic.

We show the cohort changes in marriage timing for Chinese men and women in Table 1. The median marriage age increased from 23.3 for men born before 1950 to 26.0 for men born between 1985 and 1989. A parallel increase occurred from 20.2 to 23.3 for women of the same cohorts. For both men and women born before 1970, we observe an early and universal pattern of marriage, with more than 95 percent getting married before reaching the age of 30. Among the cohorts born in the 1970s and 1980s, the timing of marriage entry is delayed, and the proportions remaining single after age 30 and 35 increased, particularly for men.

**Table 1:** First Marriage Timing for Chinese Men and Women by Birth Cohort

	Birth cohort					
	< 1950	1950–59	1960–69	1970–79	1980–84	1985–89
Male						
Median marriage age	23.3	24.4	23.3	24.3	25.2	26.0
Marriage rate before 25y (%)	65.8	57.8	71.5	56.4	48.3	41.6
Marriage rate before 30y ^(^%)	89.9	91.8	93.1	87.6	81.5	72.6
Marriage rate before 35y (%)	95.4	96.7	97.2	93.6	88.9	N/A
Marriage rate before 40y (%)	96.6	97.5	97.8	95.2	N/A	N/A
Women						
Median marriage age	20.2	22.6	21.9	22.4	23.1	23.3
Marriage rate before 25y (%)	89.5	81.4	88.4	80.5	74.4	68.2
Marriage rate before 30y (%)	98.0	97.0	97.3	96.5	94.2	90.9
Marriage rate before 35y (%)	99.2	98.7	99.0	98.9	97.2	N/A
Marriage rate before 40y (%)	99.5	99.1	99.3	99.1	N/A	N/A

Data source: China Family Panel Studies 2018

**Fig. 2:**
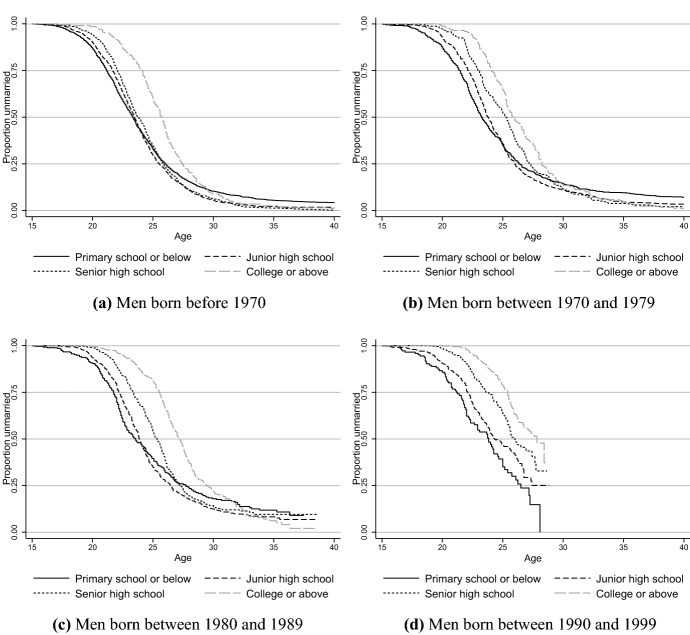
Kaplan–Meier survival curves of marriage entry for chinese men of different birth cohorts and educational levels

**Fig. 3:**
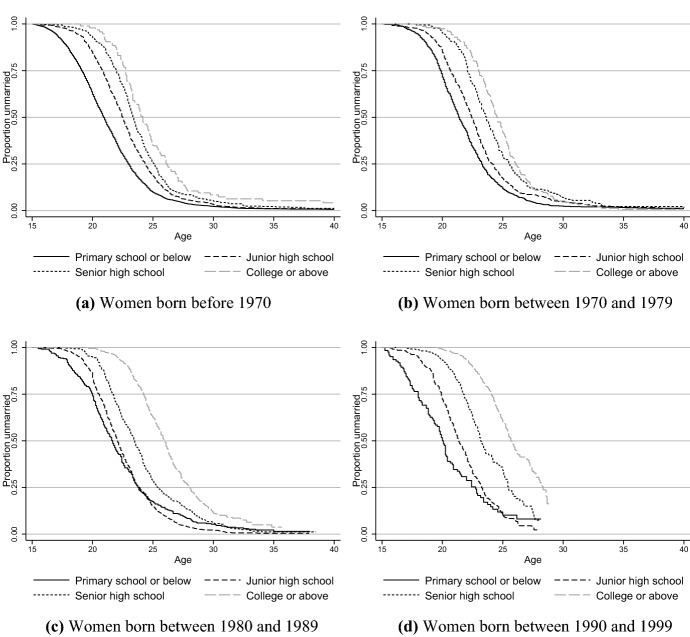
Kaplan–meier survival curves of marriage entry for chinese women of different birth cohorts and educational levels. Data source: China Family Panel Studies 2018

As found in previous studies, the economic basis for marriage has become more important among the Chinese (Ji, 2015; Yu & Xie, 2015b). In addition, the cultural tradition of female hypergamy has persisted despite the improved social status of Chinese women (Hannum, 2005; Ji, 2015; Mu & Xie, 2014; Qian & Qian, 2014). As a result, it has become more difficult for highly educated women and poorly educated men to find a matching spouse in today’s China. To better understand nonmarriage in China, we show educational gradients in marriage entry in Figs. 2 and 3. Except for those with primary school or below education, less educated men born before 1970 marry earlier than men with higher levels of education, and the life-long nonmarriage rate is less than 5 percent across all levels of education. For men born in the 1970s and 1980s, the nonmarriage rate increases for the least educated group, for whom the nonmarriage rate by age 40 increases to about 10 percent. Such nonmarried rates for these particular socioeconomic groups are unprecedentedly high in China’s modern history (Yu & Xie, 2015a). Given the increasing educational attainment and persistent hypergamy preference of Chinese women, the life-long nonmarriage rate of men with lower economic potential could continue to rise due to the mismatched SES between unmarried men and women.

Among women born before 1970, those with the highest educational level had a much higher nonmarriage rate than women of lower educational levels, with more than 10 percent remaining nonmarried by the age of 40. Such women are discriminatorily labeled as “left-over women” (*sheng nü*). However, college educated women no longer had a higher nonmarriage rate than less educated groups for those born in the 1970s, and their nonmarriage rate at age 40 approaches 0. Among the 1980s birth cohort, women with a college education tend to marry later, and a substantial proportion of them have not reached 35 years old in 2018. According to the pattern observed in women born in the 1970s, single women with the highest educational level were still at the risk of marriage entry in their 30 s. Therefore, we will not expect a higher singlehood rate for women with college education compared with those with lower educational levels among the 1980–1989 birth cohort. In other words, the phenomenon of the “left-over women” has faded out among the younger cohorts in China.

### Premarital cohabitation

We present the prevalence, duration, and consequences of premarital cohabitation in Table 2. Premarital cohabitation is relatively rare among Chinese men and women born before 1970, with fewer than 5 percent having practiced it. The premarital cohabitation rate increases among those born in the 1970s and 1980s. More than one-third of men and women born between 1985 and 1990 have cohabited before marriage. Despite its increasing prevalence, cohabitation remains a transitory union state in China. The median duration of premarital cohabitation is six months across all birth cohorts, except for those born between 1980 and 1984, for whom the median duration is nine months for men and seven months for women. The mean duration of cohabitation is about one year for both men and women of all birth cohorts. To compare changing patterns of childbearing outside of marriage, we calculate the proportion of women giving birth to children while unmarried and cohabiting. The proportion of women giving birth during cohabitation has increased slightly. For women born before 1980, about 6 percent had given birth to children while cohabiting. The proportion increased to almost 10 percent for women born in the 1980s.

**Table 2:** Prevalence and duration of and childbearing in premarital cohabitation

	< 1970	1970–79	1980–84	1985–90	1990–94
Male					
Proportion of Premarital cohabitation (%)	4.4	20.4	30.6	38.2	(22.8)
Median duration of cohabitation (months)	6	6	9	6	(6)
Mean duration of cohabitation (months)	14.9	10.1	12.9	10.8	(11.9)
Female					
Proportion of Premarital cohabitation (%)	3.3	14.9	27.5	33.9	(21.4)
Median duration of cohabitation (months)	6	6	7	6	(6)
Mean duration of cohabitation (months)	14.5	10.2	12.1	10.9	(11.1)
Childbearing during cohabitation (%)	6.9	5.7	9.8	9.5	(4.5)

Data source: China Family Panel Studies 2018

Note: The statistics of those born between 1990 and 1994 are in parenthesis because a large proportion of them have not begun to form families, and the validity of their statistics of cohabitation needs future observational data

**Fig. 4:**
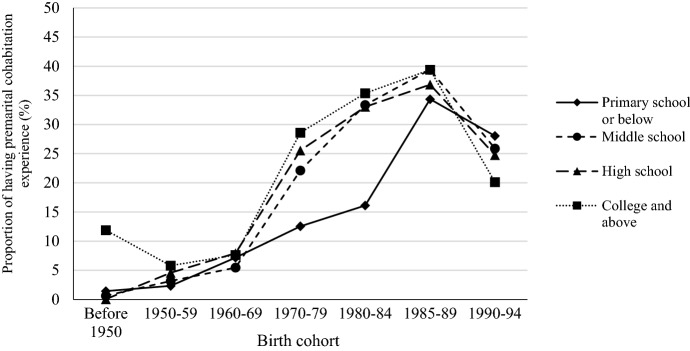
Proportion of premarital cohabitation by birth cohort and educational level for chinese men

**Fig. 5:**
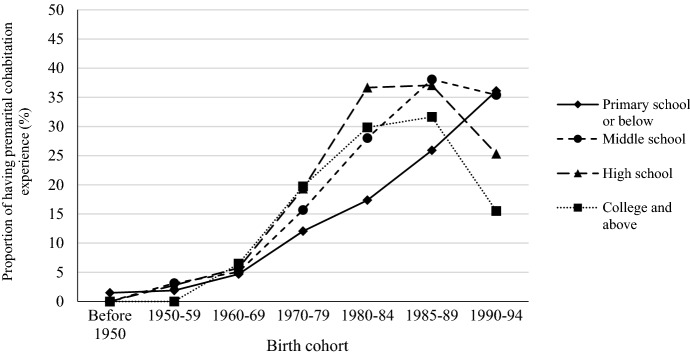
Proportion of premarital cohabitation by birth cohort and educational level for chinese women. Data sources: China Family Panel Studies 2018

As shown in previous studies, premarital cohabitation is more widely practiced among advantaged social groups in China. To capture changes in socioeconomic differentials in premarital cohabitation, we show the proportion of having premarital cohabitation experience by birth cohort and educational level men and women in Figs. 4 and 5. Among women born before 1980, those with a college degree have a higher premarital cohabitation rate than those with a lower educational level. The pattern has changes for women born in the 1980s and early 1990s. The cohabitation rate increases more quickly among women with middle school education for those born in the 1980s, and surpassed that of women with high school and college education in the late 1980 birth cohort. For those born before 1985, cohabitation is more prevalence among men with a college degree. In the 1985–1989 male birth cohort, we can observe a higher growth rate in cohabitation among those with lower educational levels, and the cohabitation rate for men with a middle school education catches up with college educated men. For men and women born between 1990 and 1994, the educational gradient has been reversed. However, since a large proportion of these young men and women have not yet begun to form families, whether cohabitation will follow the pattern of disadvantage can only be answered with future data.

### Divorce

**Fig. 6:**
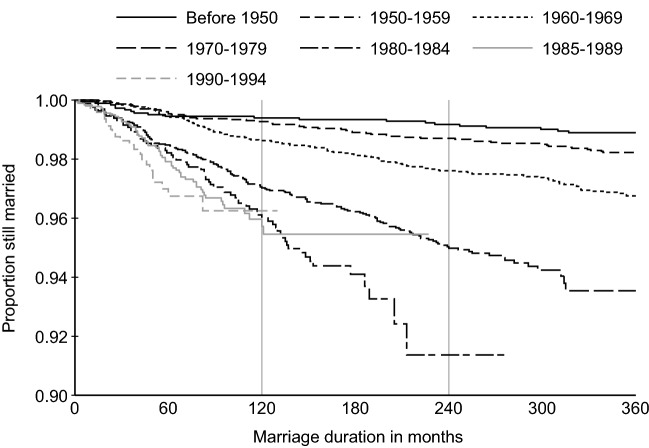
Kaplan–Meier survival curves of divorce for different birth cohorts. Data sources: China Family Panel Studies 2018

Figure 6 shows the Kaplan–Meier survival curves of divorce by birth cohort based on the 2018 CFPS data. We observe that men and women born before 1970 have stable marriages, as their cumulative divorce rate within 30 years of marriage is less than 4 percent. For those born in the 1970s, the risk of divorce is higher than for previous birth cohort, with the divorce rate exceeding 5 percent within 20 years of marriage. For the 1980–84 and 1985–89 birth cohorts, the marriage survival rate further declines, with the divorce rate at about 4 percent within ten years of marriage. Marital dissolution is further accelerated among the youngest cohort born between 1990 and 1994. However, by international standards, Chinese marriages are relatively stable. For example, the above duration-specific divorce rate (i.e., 5 percent within 10 years of marriage for those born in the 1970s) in China is lower than in the United States and other East Asian countries (Kennedy & Ruggles, 2014; Raymo et al., 2013).

**Fig. 7:**
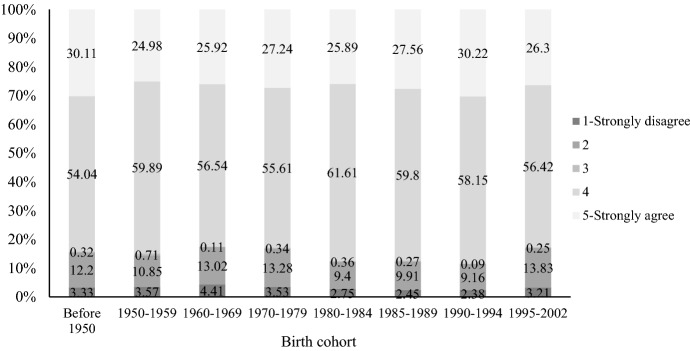
Attitudes toward “Divorce is harmful to children” by birth cohort. Data sources: China Family Panel Studies 2020

**Fig. 8:**
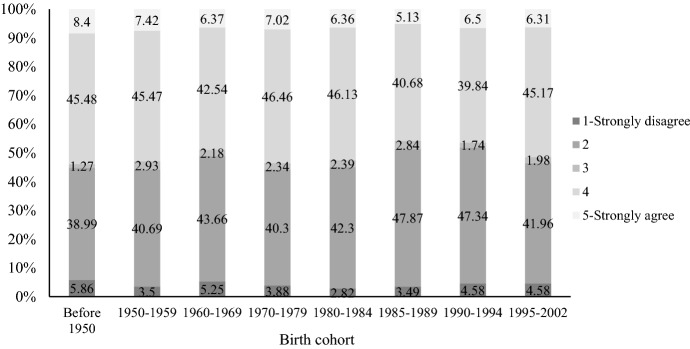
Attitudes toward “For the sake of the children, parents should not divorce despite an unhappy marriage” by birth cohort. Data sources: China Family Panel Studies 2020

One of the major reasons that marriage remains relatively stable in China has to do with attitudes toward raising children (Ma et al., 2019). In Figs. 7 and 8, we show Chinese parents’ attitudes toward divorce and childrearing based on 2020 CFPS data. Across all birth cohorts, more than 80 percent of Chinese parents agree that “divorce is harmful to children,” and we do not observe substantial changes in this attitude over time. As shown by Fig. 6, about half of Chinese parents agree that “for the sake of the children, parents should not divorce despite an unhappy marriage.” The level of agreement with this statement among parents born between 1985 and 1994 has declined, but only by a modest degree.

**Fig. 9:**
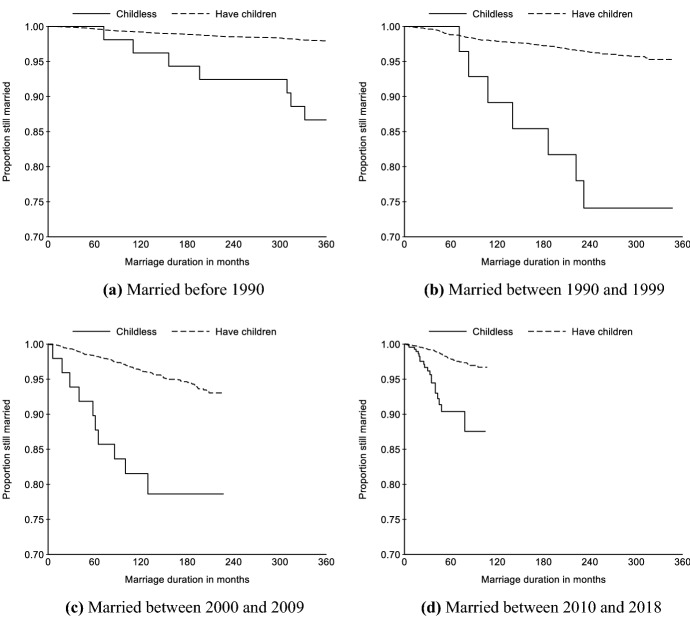
Kaplan–Meier survival curves of divorce for people of different marriage cohorts and childbearing status. Data sources: China Family Panel Studies 2018

In order to know the extent to which Chinese parents put into practice these beliefs regarding protecting children from broken families, we show the Kaplan–Meier survival curves of divorce by having children or not in Fig. 9. Among those married before 1990, the divorce rate among childless couples is slightly lower than among couples with children within 35 years of marriage. However, the marital dissolution rate of childless couples greatly exceeds that of couples with children among those married after 1990. For the 1990–99 marriage cohort, more than 95 percent of the marriages remain intact after 20 years among the couples with children, while about one-quarter of the couples without children were divorced within the same duration. Likewise, among the couples married in the 2000s, the 10-year divorce rate is more than 20 percent for the couples without children, compared to only about 3 percent for the couples with children. For the 2010–18 marriage cohort, the gap in marital stability between couples with and without children has persisted.

### Nonmarital fertility

**Fig. 10:**
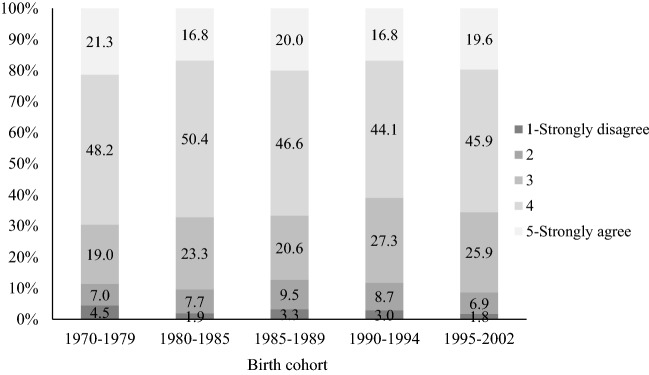
Attitudes toward “single women have the right to give birth to children” by birth cohort among Chinese women. Data sources: Intention and Behavior of Childbearing and Parenting Survey of China

Influenced by Confucianism, unmarried women who gave birth to children were traditionally considered morally corrupt, and were subject to discrimination in China (Hemminki et al., 2005). Moreover, the family planning policies of the People’s Republic of China stipulated that unmarried women had to pay a large social compensation fee for nonmarital childbirth, and they often encounter difficulties in securing public services for children born out of wedlock, such as *hukou* registration, school enrollment, healthcare, and other aspects of social welfare. In order to measure changes in the degree of stigmatization of unmarried mothers, we present attitudes toward the statement “single women have the right to give birth to children” using CIBCPS data in Fig. 10. Overall, Chinese people were supportive of single women’s right to have children, with fewer than 15 percent holding negative attitudes (“disagree” and “strongly disagree”) across all birth cohorts. We do not observe substantial variations in the attitudes toward single women’s right to have children across cohorts. Our results suggest a relatively liberal atmosphere for nonmarital childbearing in China.

To compare ideational and behavioral changes relating to nonmarital childbearing, we show the premarital conception rate, premarital childbirth rate, and marriage rate after premarital childbirth among women who have ever given birth to children using 2016 CFPS data in Table 3. Due to the lack of direct questions about premarital conception in CFPS, we construct a proxy by comparing the eldest child’s birth date and the mother’s marriage date. If the gap between a woman’s first childbirth and first marriage is less than nine months, she is defined as having a premarital conception. As premarital sexual behavior and premarital cohabitation have become widely practiced and accepted in China, the premarital conception rate has increased across birth cohorts. For the 1985–1989 birth cohort, about one-third of women who have ever given birth to children were pregnant before marriage. The premarital conception increases to 40.8 percent for mothers born between 1990 and 1994. However, since a substantial proportion of the youngest cohorts have not married yet, it will take more time to see if the premarital conception rate has declined recently.

**Table 3:** Proportion of premarital conception and childbirth among chinese women

Birth cohort	Proportion of premarital conception among all women	Premarital childbirth				
		Proportion of premarital childbirth among all women	% of marry within 1 year after childbirth among women with premarital childbirth	% of marry within 3 years after childbirth among women with premarital childbirth	% of marry within 5 years after childbirth among women with premarital childbirth	% of marry within 12 years after childbirth among women with premarital childbirth
Before 1950	7.8	4.2	35.4	67.9	71.0	89.2
1950–1959	14.3	4.8	53.5	74.2	81.9	93.5
1960–1969	20.3	5.8	61.9	78.0	83.8	92.5
1970–1979	21.9	5.3	60.6	69.3	76.2	95.5
1980–1984	25.9	7.5	55.2	76.4	83.1	91.1
1985–1989	35.8	6.9	59.6	76.6	82.0	91.9
1990–1994	(40.8)	(6.5)	(46.7)	(73.0)	(73.0)	(77.9)

Data sources: China Family Panel Studies 2016

Note: The statistics of those born between 1990 and 1994 are in parenthesis because a large proportion of them have not married, and future observational data is needed to validate their statistics of premarital conception

Despite the fact that more Chinese women are experiencing pregnancy before marriage, the majority of them are getting married before childbirth. The nonmarital childbirth rate increased from 4.2 percent among those born before 1950 to about 8 percent for those born in the 1980s. We also present the rates of getting married after premarital childbirth in Table 3. We can see that more than half of women who gave birth to a child before marriage got married within one year after childbirth. Within three years after a premarital childbirth, more than two-thirds of women had entered marriage. Except for the youngest cohort born between 1990 and 1994, about 95 percent of women having a premarital childbirth got married within 12 years after the birth. In other words, very few Chinese children grow up in nonmarital families. In contrast to the high degree of tolerance toward unmarried mothers, raising children in a nonmarital setting remains uncommon for women in China.

### Childlessness

**Fig. 11:**
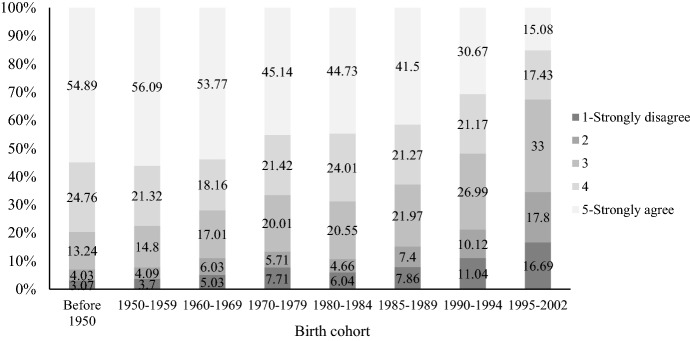
Attitudes toward “motherhood makes a woman complete” by birth cohort among Chinese men

**Fig. 12:**
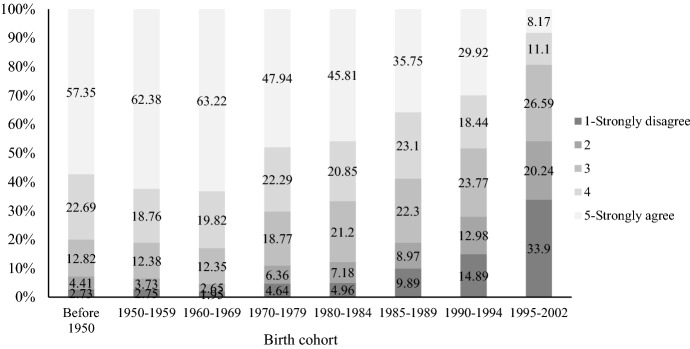
Attitudes toward “motherhood makes a woman complete” by birth cohort among chinese women. Data sources: China Family Panel Studies 2020

Childlessness is a key indicator of the SDT, as it implies a shift away from the child-centered model of the family (Zaidi & Morgan, 2017). Scholars have observed increased voluntary childlessness in many societies experiencing the SDT (Merz & Liefbroer, 2012; Potârcă et al., 2013; Rosero-Bixby et al., 2009; Rowland, 2007; Sobotka et al., 2003). Along with the declining fertility rate, the discussion of childlessness in social media has been growing tremendously in China, especially among well-educated young women (Yu, 2021). Figures 11 and 12 shows attitudes toward women’s childlessness by gender and birth cohort using 2020 CFPS data. For both men and women, we observe increasing tolerance toward childlessness, especially among those born after 1990. In addition, the changes are more pronounced among women. For the youngest women born between 1995 and 2002, less than 20 percent agree that “motherhood makes a woman complete,” and more than one-third strongly disagree.

**Fig. 13:**
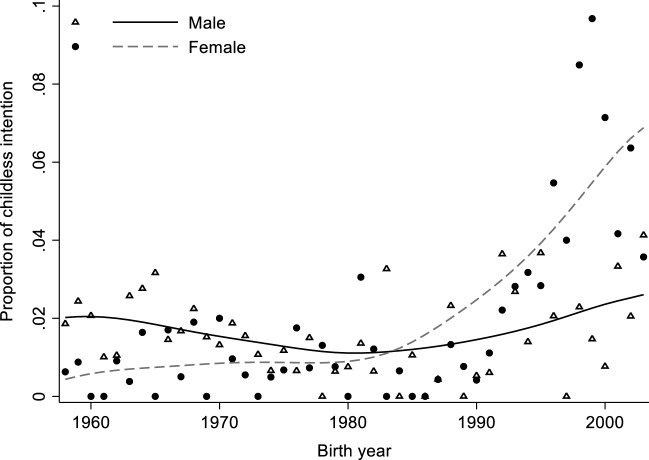
Proportion of Childlessness Intention by Birth Year for Chinese Men and Women. Data sources: China Family Panel Studies 2018

In addition to ideational changes toward childlessness, we further explore the proportion of Chinese prepared to be childless. For those whose ideal number of children is zero, we consider them to have the intention of remaining childless, and the results are shown in Fig. 13. For men, childlessness intention remains at a low level, lower than 4 percent across all birth cohorts. In contrast, we observe a more prominent increase in the proportion of women who intend to be childless. The growth is relatively slower among individuals born between 1960 and 1980, but it accelerates among the cohorts born after 1980. For women born in the late 1990s, nearly 10 percent intend to be childless.

Using 2018 CFPS data, we show childlessness rates by birth cohorts in Table 4. Among the earlier birth cohorts, childlessness is rare among women, particularly among married women. For women born in the 1960s and 1970s, only about 1 percent have not given birth to children. Compared to other East Asian societies such as Japan and Hong Kong, where about 30 percent of women of the same birth cohorts are permanently childless (Sobotka, 2021), the level of childlessness remains extremely low in China. Among the younger women born after 1980, we can observe an increase in the childlessness rate. However, the younger women are still of reproductive age, it will take longer to arrive at a conclusion.

**Table 4:** Childlessness rate among chinese women

Birth cohort	% of Childlessness among all women	% of Childlessness among ever married women	% of voluntary childlessness (Ideal = 0, child = 0)	Type of childlessness		
				% of involuntary childlessness (Ideal > 0, child = 0)	Total	N
Before 1950	2.4	2.2	12.4	87.6	100.0	23
1950–1959	2.7	2.6	2.4	97.6	100.0	43
1960–1969	1.1	0.7	41.7	58.4	100.0	25
1970–1979	1.8	1.0	16.5	83.5	100.0	38
1980–1984	8.12	5.6	11.4	88.6	100.0	67
1985–1989	17.4	9.8	2.0	98.0	100.0	167
1990–1994	(65.0)	(31.7)	(6.0)	(94.0)	100.0	519

Data sources: China Family Panel Studies 2018

Note: The statistics of those born between 1990 and 1994 are in parenthesis because a large proportion of them have not married or planned to have children, and future observational data is needed to validate their statistics of childlessness

Comparing childbearing status and the ideal number of children, we divide childless women into two types: the voluntarily and involuntarily childless. We define voluntarily childless women as those who have never given birth to a child and whose ideal number of children is zero; the others are defined as involuntarily childless. Except for the 1960–1969 birth cohort, most childless women were involuntarily childless. Although a large proportion of the post-1990s cohort are not yet married and remain of childbearing age, fewer than 10 percent of them voluntarily intend to be childless. Taken together, we do not expect a sharp rise in the childlessness rate in China in the short run, and thus we do not expect childlessness to reach the level of other East Asian societies.

## Summary and discussion

In the past two decades, the SDT has been one of the primary theoretical frameworks for understanding sustained below-replacement fertility and new family behaviors. While scholars have studied the SDT in Western and Latin American countries, East Asia has seldom been included in the comparative literature. As a Confucian society that has experienced dramatic economic and social changes in recent decades, China provides us with an opportunity to consider how modernization and ideational changes have influenced a traditional family system. Capitalizing on census, nationally representative survey, and online survey data, we provide estimates of major ideational and behavioral indicators of the SDT in China.

Our results reveal a postponement of the first marriage age for both men and women. For the population as a whole, the nonmarriage rate is low; however, men with lower educational levels have been increasingly excluded from marriage. In contrast, the so-called “left-over women” phenomenon appears to be losing relevance. With the growing cost of living and intensified competition in today’s China, women with better socioeconomic prospects have become more attractive in the marriage market, consistent with the proposition of “marriage search theory” that emphasizes the importance of women’s financial capacity (Oppenheimer, 1988). Therefore, we expect a positive educational gradient in Chinese women’s marriage timing among the younger generations in the future.

While premarital cohabitation is on the rise our results show that most premarital cohabiting unions last for a relatively short period. Despite its commonness as a practice, cohabitation is still a prelude to marriage rather than an alternative to marriage. We observe a slight increase in childbearing during cohabitation, but childbearing predominantly remains limited to the formal institution of marriage. While cohabitation used to be more prevalent among the highly educated groups, the pattern has been changed among those born in the late 1980s and 1990s. As the economic foundation of marriage keeps rising in China, there is a possibility that cohabitation could become a fallback option for people with lower SES.

In contrast with the US and other East Asian countries, the divorce rate has remained low in China. Concern for children’s development is one of the most important reasons for the stability of marriages. The vast majority of Chinese parents believe that divorce is harmful to children, and almost half of them agree that for the sake of children one should remain in an unhappy marriage rather than divorce. Such attitudes toward divorce are also confirmed at the behavioral level. Compared to couples without children, the divorce rate of couples with children is much lower for all marriage cohorts. It is worth noting, however, that the measurement of people’s attitudes in social surveys is often influenced by social acquiescence and suffer from a bias. Considering the social desirability, respondents sometimes prefer to conform with the mainstream opinion. Previous studies have shown that behavioral changes sometimes precede ideational changes in family aspect, such as fertility (Sobotka & Beaujouan, 2014). Thus, the persistence conservation attitudes towards divorce does not necessarily imply no further changes in divorce in China.

Ideologically, Chinese people are relatively tolerant of nonmarital childbearing, and about two-thirds agree that single women have the right to bear children. Due to the prevalence of premarital cohabitation, we observe an increase in the premarital conception rate. However, the premarital childbirth rate has remained relatively low and stable over time. In addition, about half of women who give birth outside of marriage subsequently get married within one year, and more than two-thirds marry within three years. In other words, very few children are raised in nonmarital families in China, and marriage is still the main institution for childbearing.

Despite the rise of anti-natalism in online discussions, we do not observe a strong tendency toward voluntary childlessness among Chinese people. The results indicate only a slight increase in childlessness intention. We also observe more tolerant attitudes toward childless women in China, particularly among young women born after 1995. Moreover, the vast majority of childlessness in China is involuntary. Among younger women born after 1990 who have not given birth to children, less than 10 percent voluntarily choose to be childless. Such findings highlight the persistent child-centered family culture in China, deeply influenced by Confucianism, which values the continuity of the family lineage. Given the ultra-low divorce rate among couples with children and the prevalence of childbearing intention among those in marriage, we expect that the divorce rate in China will not reach the level of the typical SDT countries. Limited by the information of the CFPS, we are only able to divide voluntary and involuntary childlessness by comparing the ideal number of children and childbearing status. With more detailed data on women’s fertility-related behaviors, voluntary, involuntary, and temporary childlessness can be better measured by contraceptive use, a strategy that has been frequently used in previous studies (Poston & Cruz, 2016).

Our analysis suggests that typically coincident changes in patterns of family behavior associated with the SDT are not occurring simultaneously in China. In regard to union formation, we observe more profound changes, including delayed marriage age, increased singlehood rate, and increased premarital cohabitation rate. Moreover, the socioeconomic differentials in marriage increasingly resemble those in other countries experiencing the SDT, where women’s economic prospects are gaining more importance. Premarital cohabitation is also gradually changing from an avant-garde behavior primarily practiced by highly educated people to a fallback choice for the disadvantaged who have not accumulated enough economic resources for marriage.

Taken together, our findings lend support to the proposition that there might be a distinctive pattern of the SDT in China. Although we are not positioned to generalize our results to other countries, we invite future research to examine whether the pattern of family changes in China could be generalized to other East Asian societies influenced by Confucianism and familism by studying dissimilarities and differences across East Asian societies and between East Asian societies and Western societies. Therefore, we argue that the pace and magnitude of the SDT may vary significantly with social contexts, especially in a society like China, where rapid modernization and a traditional family culture are coexistent.
